# Efficacy and Effectiveness of Extended Nursing Roles in Primary Dementia Care ‐ Results of the Multicenter, Cluster‐randomized Trial

**DOI:** 10.1002/alz70858_104268

**Published:** 2025-12-26

**Authors:** Maresa Buchholz, Bernhard Michalowsky, Anika Raedke, Moritz Platen, Annelie Scharf, Fabian Kleinke, Neeltje van den Berg, Wolfgang Hoffmann

**Affiliations:** ^1^ German Center for Neurodegenerative Diseases e.V. (DZNE), Site Rostock/Greifswald, Greifswald, Germany; ^2^ Deutsches Zentrum für Neurodegenerative Erkrankungen e. V. (DZNE), site Rostock / Greifswald, Greifswald, Mecklenburg‐Vorpommern, Germany; ^3^ Deutsches Zentrum für Neurodegenerative Erkrankungen, Rostock/Greifswald, Germany; ^4^ Deutsches Zentrum für Neurodegenerative Erkrankungen e.V. (DZNE), Rostock/Greifswald, Germany; ^5^ Institut for Community Medicine, University Medicine, University of Greifswald, Greifswald, Germany; ^6^ Institute for Community Medicine / University of Greifswald, Greifswald, Germany

## Abstract

**Background:**

Extended nursing roles in dementia care can improve treatment, care, and patient and caregiver outcomes. We tested the efficacy and cost‐effectiveness in People living with Dementia (PlwD) and caregivers.

**Method:**

We analyzed data from 417 PlwD within the multicenter, cluster‐randomized InDePendent trial six months after baseline. Specifically qualified nurses carried out advanced dementia care management tailored to patient's and caregiver's needs. Outcomes includes unmet needs (CANE), Quality of Life (QoL‐AD, EQ‐5D), Caregiver burden (Zarit), and cost‐effectiveness (FIMA, RUD).

**Result:**

PlwD receiving the intervention had 74% lower unmet needs (0.26, CI^95%^0.17‐0.40, *p* <0.001) and higher quality of life (0.04, CI^95%^ 0.01–0.07, *p* = 0.017) than controls. There was a gain in QALY (0.01, CI^95%^‐0.001‐0.018) at slightly higher costs (1,425€, CI^95%^ 638‐2,211). There was no effect on caregiver burden.

**Conclusion:**

The results provide evidence for the efficacy and effectiveness of extended nursing roles in dementia care, demonstrating that extended nursing roles reduce unmet needs and improve quality of life.

